# Gender difference in knowledge of tuberculosis and associated health-care seeking behaviors: a cross-sectional study in a rural area of China

**DOI:** 10.1186/1471-2458-8-354

**Published:** 2008-10-08

**Authors:** Jianming Wang, Yang Fei, Hongbing Shen, Biao Xu

**Affiliations:** 1 Department of Epidemiology, School of Public Health, Fudan University, Shanghai, PR China; 2 Department of Epidemiology and Biostatistics, School of Public Health, Nanjing Medical University, Nanjing, PR China

## Abstract

**Background:**

Tuberculosis (TB) detection under the national TB control program in China follows passive case-finding guidelines, which could be influenced by the accessibility of health service and patient's health-care seeking behaviors. One intriguing topic is the correlation between men and women's knowledge on TB and their health-care seeking behaviors.

**Methods:**

Two cross-sectional studies were separately carried out in Yangzhong County, a rural area of China. One study, by using systematic sampling method, including 1,200 subjects, was conducted to investigate the TB knowledge among general population. Another study in the same source population screened 33,549 people aged 15 years or over among 20 stratified cluster-sampled villages for identifying prolonged cough patients at households and individual interviews were then carried out. Gender difference in the knowledge of TB and health-care seeking behaviors was analyzed particularly.

**Results:**

Among general population, only 16.0% (men 17.1% vs. women 15.0%) knew the prolonged cough with the duration of 3 weeks or longer was a symptom for suspicious TB. Fewer women than men knew the local appointed health facility for TB diagnosis and treatment as well as the current free TB service policy. Moreover, women were less likely to learn information about TB and share it with others on their own initiatives. On the contrary, after the onset of the prolonged cough, women (79.2%) were more likely to seek health-care than men (58.6%) did. However, a large part of women preferred to visit the lower level non-hospital health facilities at first such as village clinics and drugstores.

**Conclusion:**

TB and DOTS program were not well known by rural Chinese. Gender issues should be considered to reduce diagnostic delay of TB and improve both men and women's access to qualified health facility for TB care. Strengthening awareness of TB and improving the accessibility of health-care service is essential in TB control strategy, especially under the current vertical TB control system.

## Background

Tuberculosis (TB) is a leading cause of death world-wide, especially in low-income and middle-income countries [1]. Although TB prevalence and death rates have probably been falling globally for several years, the total number of new cases is still rising slowly, due to the case-load continuing to grow in the African, Eastern Mediterranean and South-East Asia regions [2].

China has the world's second largest number of TB cases [3]. To fight against TB, the Chinese National TB Control Program (NTP) has adopted the directly observed treatment, short course (DOTS) strategy since 1992 [4]. However, the progress in TB control was slow during the 1990s, resulting in the detection rate of TB stagnating around at 30%, far below the target set by World Health Organization (WHO) [3]. Recently, especially after the outbreak of Severe Acute Respiratory Syndrome (SARS) in 2003, the Chinese government has taken a series of measures to strengthen its public health system and put great efforts on TB control. However, as a country with large populations, China is still facing great challenges, especially in rural areas. One of them is the accessibility of TB services toward the entire population [5,6]. Although China's NTP has set a free TB service policy, in most places access to TB care is still unsatisfactory.

TB control system in China is vertically composed by specialized TB dispensaries and TB control departments from county/district level to national TB Center. The basic unit of TB control in rural China is the county TB dispensary which is the main place for DOTS implementation. As case detection in the NTP in China follows WHO recommended passive case-finding guidelines, people with TB related symptoms should be identified when they seek care at a general health facility, and referred to the specialized TB dispensary for diagnosis, treatment and case management. Therefore, early detection of TB depends on whether patients could perceive their needs of seeking health-care for TB symptoms such as cough; and whether patients could be promptly referred to TB dispensaries by doctors in general hospitals and other health providers [7]. However, under the current fee for service and bonus-related revenue mechanism in China's health system, it is not surprising to find that the referral does not work well in many places [4]. Thus, making people understand when and where they should seek health-care is of great importance. Several studies have proved that lack of knowledge to TB is likely to hinder positive health-care seeking behavior whilst better knowledgeable on TB was significantly related to health-care seeking action [8-10]. Studies also found that there was gender difference in knowing TB. As reported by Agboatwalla in Pakistan and Shetty in London, knowledge of TB was generally deficient in women, particularly in rural women [11,12].

Gender disparity is focused world-wide as higher notification rates of TB among men than women have been observed in many countries [13]. These findings raise the hypothesis that TB among women might be under-reported in developing countries. It has been supported by the results from several studies comparing active and passive case-finding strategies [14]. One study in Bangladesh reported that women, in comparison with men, had significantly longer diagnostic delay and patient delay [15]. Similar results could be found in Shandong Province of China, where women experienced longer health system delays than men, and that the higher the level of health facility patients first visited, the less time was needed to achieve a diagnosis [16]. Our former qualitative study in China also found a gender disparity in the experiences of health-care seeking and access to TB care [17]. Factors affecting patient's behavior were complex. Whether the gender difference in health-care seeking behavior is associated with the disparity of knowledge to TB among men and women is unclear. Few studies have been focused on this issue.

The purpose of the present study was to understand whether and what extent people in rural China know TB and aware of the pro-poor DOTS program, and further to understand the collation between rural people's knowledge and awareness of TB and their health-care seeking behaviors from a gender perspective.

## Methods

### Study site

This study was conducted in YZ County, an island locating on the middle of Yangtze River in the southeast part of China, with a population of about 0.3 million and an area of about 332 km^2^. This is a relatively rich area ranked as one of the 100 richest counties in China. The county TB dispensary is affiliated to CDC (Center for Disease Control and Prevention), which was formerly called Anti-Epidemic Station. It is the exclusive appointed health facility responsible for TB diagnosis and treatment for the county residents (County hospital is appointed for severe inpatients). All suspected TB patients should be referred to this unit for further examination. Free diagnosis and treatment are available in TB dispensary for sputum smear positive patients (it has been expanded to all patients including sputum smear negative patients since 2005). Here, 'free' means no charges for sputum smear test, chest X-ray examination and anti-tuberculosis medications distributed by government. All other health facilities in this county including township health centers, private practitioners and village health stations are responsible for referring TB suspects to the county TB dispensary, and smear microscopy tests and anti-tuberculosis medicines are not available in these facilities.

### Study design and data collection

Two cross-sectional studies were separately conducted in the study site.

#### (1) Knowledge on TB among general population

Sampling strategy in the current study followed the guidelines designed by China CDC. After sorting all towns in YZ County by socioeconomic status (gross domestic product), 2 towns were selected at the first stage by using a systematic sampling technique. Then 3 villages from each town were systematically sampled. At the third stage, 100 households were systematically sampled from each village based on the list of householders' names. In each household, two family members (aged 12 to 65) whose birthday (month and day) was close to the investigation date (month and day) were selected as study subjects and were then interviewed by trained investigators with a detailed questionnaire. This questionnaire we adopted in the study referred to the questionnaire designed by China CDC, which has been applied in a national survey on the knowledge, attitudes and practices (KAP) towards TB in China [18].

#### (2) Health-care seeking behaviors among TB suspects

Among the same source population, a stratified cluster sampling method was used to select sample units for TB screening. Totally, 20 villages were randomly sampled from 5 towns (one town named as SM where the county center located was not involved in the sampling process). All permanent inhabitants aged 15 years or over were the study population and then screened by using a simple questionnaire for identifying people at each household with prolonged cough which was a main symptom for TB. A detailed structured questionnaire was then administered for all identified cough cases to collect socioeconomic and demographic variables, symptoms other than cough and health-care seeking behaviors. These patients with prolonged cough were regarded as TB suspects and were then referred to CDC for free X-ray examination and sputum smear microscopy test.

In this study, delays in TB diagnosis are generally divided into 'patient delay" and 'system or service provider delay'. 'Patient delay' refers to the time between the first onset of symptoms and first utilization of a healthcare provider, whilst 'system delay' refers to the time between the first utilization of a health provider and a confirmed diagnosis of TB [7].

### Data analysis

Data were analyzed by SPSS 11.0 software (Chicago, Illinois, USA). Chi-square test for proportions and student's *t*-test and Kruskal-Wallis H test for continuous variables were used to describe differences between groups. About 10% of all cases were randomly selected to be re-interviewed through telephone by the supervisor after field investigation. In this study, a prolonged cough was defined as the cough lasting for 3 weeks or longer. Health-care seeking behaviors included buying drugs in pharmacies and visiting private practitioner, village health workers, physicians in town, county or upper level hospitals and the County TB Dispensary. Formal health-care seeking was exclusively defined as the experience of visiting town or upper level hospitals. Health-care seeking delay referred to a period from the onset of symptoms to the first utilization of a health facility.

### Ethical consideration

Oral inform consent was obtained in the study on the knowledge of TB among general population. Written inform consent was obtained from all participants in the study on health-care seeking behavior among TB suspects. The study was approved by Institutional Review Board in School of Public Health, Fudan University.

## Results

### 1. Knowledge on TB among general population

One thousand and two hundred adults were selected for the survey of knowledge on TB and 1083 subjects completed the questionnaire. The proportion of men and women were 46.4% and 53.6% respectively, with the average age of 43.1 ± 12.9 years. The median annual income per capita was around 4000 CNY (Chinese Yuan). As shown in table 1, 99.2% of the subjects have heard about TB and a large part of them regarded it as a contagious disease (men 92.6%; women 91.6%). Many of them thought TB was a relatively severe disease, which could influence the labor ability. About 15.6% of them actively acquired information about TB and 13.1% of them shared it with others on their own initiatives. Significantly more men than women actively learned knowledge about TB (men 20.1% vs. women 11.7%, P < 0.001). Sixteen percent of them (men 17.1% vs. women 15.0%) understood that the prolonged cough with the duration over 3 weeks was a suspicious symptom for TB. When inquired about the current TB policy in YZ County, 63.4% (men 69.9% vs. women 57.8%, P < 0.001) answered that they knew about the appointed health facility for TB diagnosis and treatment. Less women than men knew the local policy for free TB service with a significant gender disparity. Approximately 38.6% women vs. 46.8% men (P = 0.007) knew that it was free for TB diagnosis as well as 34.4% women vs. 44.6% men (P < 0.001) knew that it was free for TB treatment in the local county. Only 73.6% (men 78.5% vs. women 69.4%) believed TB was a curable disease at the present time.

**Table 1:** Responses to questions towards TB among men and women in a rural area of China

Questions		Total(n = 1083) n (%)	Men(n = 502) n (%)	Women(n = 581) n (%)
1. Have you ever heard about TB?	No	9(0.8)	3(0.6)	6(1.0)
	Yes	1074(99.2)	499(99.4)	575(99.0)
				
2. Is TB transmissible?	Don't know	55(5.1)	22(4.4)	33(5.7)
	No	31(2.9)	15(3.0)	16(2.8)
	Yes	997(92.1)	465(92.6)	532(91.6)
				
3. Do you regard TB as a severe disease?	Don't know	44(4.1)	20(4.0)	24(4.1)
	No	11(1.0)	9(1.8)	2(0.3)
	Severe	250(23.1)	113(22.5)	137(23.6)
	Very severe	778(71.8)	360(71.7)	418(71.9)
				
4. Do you think TB will influence the labor ability?	Don't know	43(4.0)	17(3.4)	26(4.5)
	No	9(0.8)	4(0.8)	5(0.9)
	Yes, but little	294(27.1)	132(26.3)	162(27.9)
	Yes, seriously	737(68.1)	349(69.5)	388(66.8)
				
5. Did you actively learn something about TB?	No	914(84.4)	401(79.9)	513(88.3)
	Yes	169(15.6)	101(20.1)	68(11.7)
				
6. Did you actively share the knowledge of TB with others?	No	941(86.9)	429(85.5)	512(88.1)
	Yes	142(13.1)	73(14.5)	69(11.9)
				
7. What's the duration of cough regarded as TB suspicious symptom?	Don't know	490(45.2)	196(39.0)	294(50.6)
	<1 week	75(6.9)	41(8.2)	34(5.9)
	1 week+	129(11.9)	87(17.3)	42(7.2)
	3 week+	173(16.0)	86(17.1)	87(15.0)
	1 month+	216(19.9)	92(18.3)	124(21.3)
				
8. Is there an appointed health facility for TB diagnosis and treatment?	Don't know	377(34.8)	142(28.3)	235(40.4)
	No	19(1.8)	9(1.8)	10(1.7)
	Yes	687(63.4)	351(69.9)	336(57.8)
				
9. Is it free for TB diagnosis in your county?	Don't know	484(44.7)	209(41.6)	275(47.3)
	No	140(12.9)	58(11.6)	82(14.1)
	Yes	459(42.4)	235(46.8)	224(38.6)
				
10. Is it free for TB treatment in your county?	Don't know	463(42.8)	201(40.0)	262(45.1)
	No	196(18.1)	77(15.3)	119(20.5)
	Yes	424(39.2)	224(44.6)	200(34.4)
				
11. Do you believe TB is a curable disease?	Don't know	60(5.5)	24(4.8)	36(6.2)
	No	48(4.4)	19(3.8)	29(5.0)
	Sometimes	178(16.4)	65(12.9)	113(19.4)
	Yes	797(73.6)	394(78.5)	403(69.4)

### 2. Health-care seeking behaviors among TB suspects

By screening 33,549 people (16,227 men and 17,322 women), 190 subjects were notified with a prolonged cough within the past three months. After recheck, 7 patients were excluded due to the short durations of cough. Another 12 former TB patients diagnosed three months ago were also excluded. Finally, 171 subjects (99 men and 72 women) identified as TB suspects were involved in the analysis. As shown in table 2 and table 3, 67.3% of them had sought for health-care during the current cough episode and only 30.4% of them went to the town hospital or upper levels seeking for formal health-care. Nearly 59.6% of them firstly visited village clinics or drugstores after the onset of cough. The median of household per capita income was 4000 CNY and 3000 CNY respectively in the group with or without seeking health-care (*P *= 0.042). More women than men sought health-care for the current prolonged cough with a significant gender difference (women 79.2% vs. men 58.6%, *P *= 0.005). However, men preferred to visit upper level health facilities first, whereas women preferred to visit lower level health facilities first (Table 3). Even in the second health-care seeking episode, this gender difference still existed. The median of delay from the onset of symptoms to the first visit at health facility was 10 days. There was no gender disparity on the patient delay among TB suspects (Kruskal-Wallis test, *P *= 0.305). After screen, the identified patients with prolonged cough were referred to CDC for further free check, 128 (74.9%; men/women: 74.7%/75.0%) traveled to CDC for free chest X-ray examination and 153 (89.5%; men/women: 89.9%/88.9%) provided samples for free sputum smear test. Among 153 TB suspects being further rechecked, 8 (7 men and 1 woman) were ultimately diagnosed with pulmonary TB.

**Table 2:** Relation between social economic and demographic characteristics and health-care seeking behavior

	Seeking health-care		
		
Variables	No (n = 56)	Yes (n = 115)	*P*
Gender			
Men	41	58	0.005^†^
Women	15	57	
Age (years)			
Mean ± SD	65.3 ± 14.7	63.2 ± 13.3	0.362^‡^
Household per capita income (CNY)			
Median	3000	4000	0.042*
Education (years)			
Illiterate	18	47	0.477^†^
Primary school	21	41	
Secondary school and above	17	27	
Marital status			
Single/married	46	94	0.949^†^
Divorced/bereft of spouse	10	21	

†:Chi-square test; ‡:Student's *t*-test; *:Kruskal-Wallis H test

**Table 3:** Level of health facilities TB suspects visited during the first two health-care seeking*

	1st visit			2nd visit		
		
Level of Health facility	Total(n = 109) n (%)	Men(n = 56) n (%)	Women(n = 53) n (%)	Total(n = 34) n (%)	Men(n = 17) n (%)	Women(n = 17) n (%)
County hospital or upper level	14(12.8)	9(16.1)	5(9.4)	6(17.6)	6(35.3)	0(0)
County TB dispensary	0(0)	0(0)	0(0)	1(2.9)	0(0)	1(5.9)
Town hospital	30(27.5)	17(30.4)	13(24.5)	9(26.5)	4(23.5)	5(29.4)
Village clinic	51(46.8)	25(44.6)	26(49.1)	14(41.2)	6(35.3)	8(47.1)
Drugstore	14(12.8)	5(8.9)	9(17.0)	4(11.8)	1(5.9)	3(17.6)

*: Patients seeking health-care within 3 months preceding interview were involved

## Discussion

With the vertical TB control system, DOTS program characterized by the free TB diagnosis and anti-tuberculosis treatment is only available in TB dispensary. In rural areas, the lowest level of TB control system is the county TB dispensary where patients with cough and/or other TB symptoms do not routinely visit. In the context of China's TB control policy, it's not possible to see this system being replaced by the non-specialized health facilities in a near future. So the accessibility of DOTS in China relies on referral by doctors in general hospitals, and/or self-referral by patients. To empower patients, and to make people understand when and where they should seek health-care, Chinese government has initiated a massive education program on TB in general population, especially people living in the rural areas. One of the objectives of this education program is to help potential TB patients identify the suspicious symptoms and go to the right place for treatment in time. Either in the urban hospitals or in the remote rural health facilities (even in the village health station), there are posters on the wall, such as 'If coughed for more than 3 weeks, you are suspect for TB', 'The government provides free treatment for communicable TB' and 'Local CDC (TB dispensary) provides free service for sputum smear test, chest X-ray and anti-tuberculosis medicines'. This information also spread through other vivid and dramatic manners such as newspaper, website, television, broadcast, brochure and leaflet. People would argue that it does not sound reasonable to expect non-patients and/or potential patients to know where to go for TB diagnosis and treatment, but it's a compromise to the vertical TB control system. Theoretically, the health staff that TB patients encounter should refer them to the correct place for diagnosis and treatment, where DOTS program is available. However, this referral system does not work well in many places [5,19]. As we know, under China's health system reforms, hospitals and other health facilities have adopted fee for service and bonus-related revenue systems to encourage their medical staffs to make more money [4]. It is not surprising, therefore, that these health facilities have been developing a variety of means to attract patients in order to generate more revenues by providing more services and selling more drugs [4]. It is also common to see that, repeated outpatient visits before diagnosis, over-prescription of drugs and prolonged treatments instead of referral to appointed health facilities in time [17]. Admittedly, the heavy financial burden on TB patients is one of the major problems in China's TB control which has been the main reason for poor access to TB care and treatment compliance. Pressure to generate revenue and competence of health workers at different levels cause diagnostic delay and high economic burden to TB patients and ultimately impede effective TB control in China [20]. But, if patients know TB diagnosis and treatment should be free, they would have more chances to ask why they should pay for TB care and what cost should be covered by the free care. Therefore, on one hand, regulating doctors' referral could be effective to shorten diagnosis delay for TB; on the other hand, educating general population to seek health-care in an appropriate way is also an alternative.

Massive health education programs in China have been proved to make a great impact on the enhancement on people's knowledge about TB. From the current study, we are also glad to find that almost all people have heard about TB and more than 92% knew it was a transmissible disease. However, knowledge about TB linked with health-care seeking behaviors still seems unsatisfactory. Only 16% of them knew that cough lasting for more than 3 weeks was a suspicious symptom for TB and less than half of them knew the free policy for TB diagnosis and treatment. The incomprehensive perception on TB among general population after the massive education program arouses our consideration on the health educations in China: whether it is a successful campaign and what is the cost-effective way?

One interesting result in our study is that the gender disparity of knowledge towards TB among men and women was inconsistent with the health-care seeking behaviors. Compared with men, women lacked knowledge about TB symptoms and the pro-poor service policy. However, they were more likely than men to seek health-care after the onset of TB suspicious symptoms. As proved in several studies, deficient knowledge in women and patient's recognition of TB were statistically significant factors of diagnostic delay for TB [11,21]. A study in rural Inner Mongolia of China also reported that women with less education tended to be less knowledgeable about TB and were less likely to seek care than men though gender difference was not statistically significant in the quantitative survey [22]. In our current study, lack of knowledge among women did not show negative impacts on their health-care seeking. This phenomenon could also be found in South India that despite facing greater stigma and inconvenience, women were more likely than men to access health services and adhere to treatment [23]. However, when we take a deep look on the data and further explore their health seeking experiences, it is not surprised to find that men and women have different preference on the health-care service. Men preferred to visit upper level health facilities – the hospitals, whereas women preferred to visit lower level health facilities such as village health stations. As proved by other studies, patients who chose the village clinic or private providers as their first health facility usually experienced a much longer health system delay than that of those choosing other formal heath facilities [16,24]. Thus though women were more likely to seek health-care for TB suspicious symptoms, it might not help shorten the health system delay due to the weakness in diagnosis in non-formal health facilities. There are several explanations for this phenomenon. One might be the deficient knowledge on TB we discussed above. Another might be the special role of women in China. In rural areas of China, most work in the household is undertaken by women in addition to agricultural work, which may mean that they have less time seek health-care in a township health center or general hospital. Women may therefore prefer to visit facilities that are geographically accessible such as village health stations or private practitioners.

Another intriguing phenomenon found from this study also need to be further studied, which was that, though free service was provided to the identified cough patients, some of them were still not willing to get further examination. When inquired about the potential reasons, some patients answered "Free? I don't believe it. After examination, I am sure they will administrate many drugs and charge me a lot", and others said "That is only cough. I know it will not be a serious disease..." More reasons undermining this aspect need further studies.

One of the limitations in this study is that data were only collected from one county, which might not truly reflect the vision of the whole population in China. Though the study is very small, and findings from this study may not be comprehensive, it does have impacts on gender equity in TB control of China. Another limitation is that information depended on self-reported data and the survey on health-care seeking behavior was based on recall history. To minimize recall bias, some strategies had been taken, such as questionnaires were pre-tested and all questions were set to be easy understood; investigators were carefully trained and supervised. Ten percent of subjects were re-interviewed through telephone and the consistency was more than 95%.

## Conclusion

Findings from our study indicate that knowledge and awareness of TB are still unsatisfactory in rural Chinese population. Compared with men, women have less knowledge on the current TB service policy and reluctant to actively acquire information about TB. Though they are more likely to seek health-care after the onset of prolonged cough, women usually visit village clinics or drugstores whilst men prefer to seek health-care in upper level hospitals. Gender issues should be considered in promoting patients' health-care seeking behavior and to shorten the delay of diagnosis. Improving the accessibility of healthcare service is essential in TB control strategy, especially under the current passive case-finding guidelines. Results of this study are derived from a rural population of China, but could be discussed also in relation to other populations with the similar condition.

## Competing interests

The authors declare that they have no competing interests.

## Authors' contributions

JW and BX conceived the idea, implemented the field study and wrote the manuscript. YF participated in the design and implement of the study and statistical analysis. HS participated in data analysis and helped to draft the manuscript. All authors read and approved the final manuscript.

## Pre-publication history

The pre-publication history for this paper can be accessed here:


